# Gluten-Free Diet in Co-Existent Celiac Disease and Type 1 Diabetes Mellitus: Is It Detrimental or Beneficial to Glycemic Control, Vascular Complications, and Quality of Life?

**DOI:** 10.3390/nu15010199

**Published:** 2022-12-30

**Authors:** Ingo Eland, Lars Klieverik, Abbas Ali Mansour, Abdulbaqi Al-Toma

**Affiliations:** 1 Department of Internal Medicine, St. Antonius Hospital, 3435 CM Nieuwegein, The Netherlands; 2 Faiha Specialized Diabetes, Endocrine and Metabolism Center (FDEMC), University of Basrah, Basrah 61013, Iraq; 3 Department of Gastroenterology and Hepatology, St. Antonius Hospital, 3435 CM Nieuwegein, The Netherlands

**Keywords:** celiac disease, gluten-free diet, type 1 diabetes mellitus, HbA1c, quality of life, glycemic control

## Abstract

Celiac disease (CeD) is associated with type 1 diabetes mellitus (T1DM), and both have the same genetic background. Most patients with T1DM who develop CeD are either asymptomatic or have mild CeD-related gastrointestinal symptoms. Therefore, children affected by T1DM should undergo screening for asymptomatic CeD. The aim of this review is to highlight the influence of a gluten-free diet (GFD) on glycemic control, growth rate, microvascular complications, and quality of life in patients with T1DM and CeD. PubMed, Google Scholar, Web of Science, and Cochrane Central databases were searched. Reports reviewed were those published from 1969 to 2022 that focused on the interplay of T1DM and CeD and examined the effect of diet on glycemic control, growth rate, and quality of life. The most challenging aspect for a child with T1DM and CeD is that most GFD foods have a high glycemic index, while low glycemic index foods are recommended for T1DM. Interestingly, dietary therapy for CeD could improve the elevated HbA1c levels. Avoiding gluten added to a diabetic dietary regimen in T1DM patients might impose practical limitations and lead to important restrictions in the lifestyle of a young patient. Consequently, non-adherence to GFD in patients with T1DM and CeD is common. GFD in patients with T1DM and CeD seems to lower the incidence of micro- and macrovascular complications, but this requires further investigation. It seems that adherence to GFD in young patients with T1DM and CeD leads to regular growth and a stable body mass index without any negative effect on HbA1c or insulin requirements. Furthermore, the lipid profile and quality of life seem to have improved with the introduction of GFD.

## 1. Celiac Disease and Type 1 Diabetes Mellitus: The Association

Type 1 diabetes mellitus (T1DM), an autoimmune disease, is caused by insulin deficiency due to destruction of the insulin-producing pancreatic beta cells. Several studies have shown the importance of adequate glycemic control in avoiding long-term diabetic complications and that partial endogenous insulin production facilitates good glycemic control [1,2].

Patients with T1DM have an increased risk of developing other autoimmune disorders such as Hashimoto’s thyroiditis, Addison disease, atrophic gastritis, pernicious anemia, vitiligo, and celiac disease (CeD) [3]. These auto-immune diseases share a similar genetic background and are associated with autoantibodies that can be detected before the development of clinical manifestations. A coexisting autoimmune disorder can complicate diabetes management. Screening protocols have been advocated to detect these disorders [4].

CeD is an autoimmune disorder in genetically predisposed individuals precipitated by exposure to gluten, a protein found in wheat, rye, barley and others, resulting in a multisystem inflammation with enteropathy as a central feature. CeD is almost always restricted to human leukocyte antigen (HLA) HLA-DQ2 or DQ8 positive individuals. The association between T1DM and CeD dates back to 1969 [5].

The genetic risk factors associated with both diseases include HLA genes and non-HLA genes. Genetic factors contribute to about 50% of the risk of T1DM [6]. The genes most strongly associated with T1DM are HLA-DQ2 and DQ8. These HLA-alleles have been linked to an increased risk of CeD. As a result, T1DM patients are more likely to develop CeD [6,7].

Studies in animals support a link between gluten and T1DM. It is suggested that gluten affects the β-cells, with a possible link to the development of T1DM. Other components may exert an additional effect, such as the innate and adaptive immune systems, pro-inflammation, and gut microbiota and permeability [7,8,9,10,11,12].

Furthermore, gliadin has been shown to affect the pancreas [13], possibly by inducing inflammation [9] and β-cell stress [10]. The incidence of T1DM in mice was reduced from 64% to 15% by applying a lifelong gluten-free diet GFD [12,14].

In addition to the genetic connection between T1DM and CeD, studies suggest that higher gluten consumption may escalate the incidence of T1DM. For example, a study found a proportional increase in the risk of T1DM in the offspring of pregnant women who consumed more gluten [15]. In another study, a GFD improved insulin secretion in subjects at high risk for T1DM [16]. A small study from Denmark showed significant improvement in glycemic control following a one-year GFD in 15 children with newly diagnosed T1DM [17].

There is some evidence suggesting that a GFD might positively influence T1DM pathology, onset, and clinical course [18]. However, studies in humans are rare. There is no clear evidence that a GFD can prevent the development of CeD in patients with T1DM [19]. Such an approach is not recommended as it makes future CeD diagnosis difficult [20].

Several environmental factors were studied as precipitating factors for the development of CeD or T1DM. Enterovirus infections, specifically Coxsackie, have been linked to the development of T1DM [21,22]. Rotavirus has been linked to an increased risk of T1DM and CeD [23]. Furthermore, altered gut permeability and microbiota seem to be factors that contribute to the development of both of these two diseases [24]. See Box 1.

Box 1 **Synopsis 1.**

1. The genetic risk factors associated with CeD and T1DM include both HLA genes and non-HLA genes.

2. The genes most strongly associated with these diseases are HLA (DQ2) and (DQ8).

3. Patients with T1DM are at an increased risk to developing CeD.

4. Studies are scarce regarding the possible preventive role of GFD on the development of T1DM.

## 2. Epidemiology and Screening

The increased prevalence of CeD in patients with T1DM is due to an overlap in the genetic susceptibility to both diseases [25]. The HLA-DQ2 (in particular 2.5 and less frequently 2.2) haplotype is present in more than 90% of CeD patients and 55% of those with T1DM, compared to 20–25% of the European population. The haplotype HLA-DQ8 has also been associated risk for T1DM and CeD [25].

In Western countries, CeD affects around 1–2% of the population. In children with T1DM, the prevalence of CeD is significantly higher than in non-diabetic children. At least 10% of patients with T1DM have CeD at some point in their lives [25,26,27,28]. Different prevalence percentages were reported depending on whether individuals with positive CeD serology or biopsy-proven CeD were included. The prevalence of positive CeD serology was 14.3% [29], while the prevalence of biopsy-proven CeD ranges between 1.6 and 16.4% among people with T1DM [30,31]. In general, the risk of receiving a diagnosis of biopsy-proven CeD is higher before the age of 5 years. It has been shown that the development of autoantibodies for T1DM usually precedes that of the anti-tissue transglutaminase autoantibodies [32].

CeD is present in 3.6% of patients at T1DM onset [33,34]. One study showed that the prevalence of CeD in T1DM has clearly increased since 1994 (10.6% vs. 6.6%, *p* = 0.015), possibly related to changes in environmental factors, such as, different eating habits and viral infections [35]. Earlier and more frequent screening for CeD in T1DM and more accurate instrumental diagnoses might have positively influenced the prevalence rate.

Additionally, the prevalence of CeD is high in adults with T1DM [36]. In 16% of cases, T1DM and CeD were diagnosed within the same year, and 8% of the patients with positive CeD were subsequently diagnosed with T1DM after they tested positive with the CeD screening test. The rest of the patients had a positive screening CeD test after being diagnosed with T1DM [37].

Most patients with T1DM who develop CeD are either asymptomatic or only mildly symptomatic; less than 10% develop gastrointestinal symptoms. Therefore, children affected by T1DM should undergo screening for asymptomatic CeD. The clinical practice guidelines of the International Society for Pediatric and Adolescent Diabetes (ISPAD) in 2018 and those of the American Diabetes Association (ADA) recommend that screening for CeD be carried out at the time of T1DM diagnosis, at 2 years and 5 years after diagnosis, or sooner if symptoms develop. More frequent screening is recommended for those children with a first-degree relative with CeD [38,39]. However, there are data suggesting that both of these screening guidelines may miss early detection of CeD in a significant number of young patients. In general, there is no uniformity in the international guidelines on CeD or T1DM regarding the screening of adult patients [20,39,40,41]. Therefore, larger prospective studies are necessary to substantiate the suggestion that it would be beneficial to expand the screening guidelines for CeD to beyond 5 years post diagnosis in T1DM even in adulthood [42]. See Box 2.

Box 2 **Synopsis 2.**

1. At least 10% of patients with T1DM have CeD at some point in their lives. The prevalence ranges between 0.6% and 16.4%. Of these patients, CeD is present in 3.6% at T1DM onset, at a younger age, and in boys.

2. Most cases of CeD in T1DM patients are asymptomatic, only 10% develop gastrointestinal symptoms. Therefore, screening for CeD should not be guided purely by symptoms.

3. Screening guidelines may miss early detection of CeD in a significant number of young patients with T1DM. There are data suggesting that it would be beneficial to expand the screening guidelines for CeD to include a period beyond 5 years post diagnosis in T1DM.

## 3. Effect of Gluten Free Diet

### 3.1. Gluten Free Diet for CeD in T1DM Patients

A gluten-free diet (GFD) is the standard therapy for CeD, avoiding all foods that contain wheat, rye, barley, and oats. This diet needs to be followed strictly for the patient’s entire life live to prevent complaints (diarrhea or constipation, abdominal pain, vitamin and iron deficiencies, failure to thrive, and tiredness) and long-term complications (enteropathy-associated T-cell lymphoma, decreased bone mineral density, associated autoimmune diseases, a high risk of infertility, and mortality) [20,43,44].

An accurate diet is essential in the management of T1DM patients [45], aiming to regulate and maintain blood glucose and blood pressure within the normal range, to secure a normal lipid profile, and to achieve a healthy body weight. Consistent glycemic control is critical for reducing T1DM micro- and macrovascular complications [46]. Therefore, it is vital to advise patients concerning carbohydrate amount, type, and distribution throughout the day. The choice of foods with a low glycemic index may be vital [47].

Instituting a GFD could be a significant hurdle because many gluten-free foods have a high glycemic index. This may have an impact on glycemic measures, HbA1c, insulin requirement, lipid profiles, and even the incidence of chronic diabetic complications. A GFD added to a diabetic dietary regimen in T1DM patients might impose practical limitations and lead to considerable restrictions in the lifestyle of a young patient. Consequently, failure to adhere to GFD among patients with T1DM and CeD is common. It has been found that only about 60% of patients with T1DM and CeD were really compliant with a strict GFD, while patients with CeD alone had a compliance rate of about 78% [48,49].

We strongly recommend that patients with these two chronic conditions to be managed by an experienced dietitian. Furthermore, a multidisciplinary management approach seems necessary, involving gastroenterology, endocrinology, dieticians, and psychologists. Seen Box 3.

Box 3 **Synopsis 3.**

1. Avoiding gluten added to a diabetic dietary regimen in T1DM patients might impose practical limitations and lead to unwanted restrictions in the lifestyle of a young patient.

2. Dietary non-adherence to GFD in patients with T1DM and CeD is common.

3. We would strongly advise that an experienced dietitian guide the management of patients with the coexistence of these two chronic conditions.

### 3.2. Glycemic Control and Glycemic Index in T1DM and CeD

Results from studies were inconsistent regarding the influence of a GFD on glycemic control, insulin dosage, HbA1c, glucose excretion, and hypoglycemic episodes in patients with T1DM and CeD. One study showed no difference between pediatric T1DM patients with and without CeD [50]. Another study showed improvements in both HbA1c and body mass index [51]. See Box 4.

Box 4 **Synopsis 4.**

1. Results from studies were inconsistent regarding the influence of a GFD on glycemic control, insulin dosage, HbA1c, and hypoglycemic episodes in patients with T1DM and CeD.

2. In diabetic patients, a low glycemic index diet is often pursued to restrain glucose excursions that are associated with increased long-term risk of microvascular complications.

3. Consumption of dietary items with a low glycemic index prior to the main source of carbohydrates may improve postprandial glycemic control. Some gluten-free food items may be low in carbohydrates; hence, hypoglycemia may develop after the administration of standard (unadjusted) dosages of insulin.

Saadah et al. [52] reported that a GFD significantly improved growth and influenced diabetic control. Other authors [51,53] did not report a significant difference in insulin dosage, HbA1c, urinary glucose excretion, or the frequency of hypoglycemic episodes in pediatric patients. In adult patients, similar findings have been reported [54]. Abid et al. [55] showed that GFD in T1DM children with CeD resulted in a short-lived reduction in gastrointestinal symptoms and the frequency of severe hypoglycemic episodes, while there was no change in scores for height, weight, BMI, or the mean HbA1c before and after GFD. The average insulin requirement increased markedly.

Sildorf et al. [18] reported an interesting case of a patient who was started on GFD after a T1DM diagnosis without coexisting CeD. The patient needed less insulin and remained without exogenous insulin after 20 months. Consequently, it is thought that the GFD might prolong the remission (honeymoon) phase of diabetes.

Creanzo et al. [56] investigated, in a case–control study, whether in T1DM patients the coexistence of long-lasting CeD treated with a GFD impacts glycemic control and the incidence and severity of microvascular complications. They concluded that GFD neither worsened glycemic control nor negatively influenced the incidence of chronic microvascular complications.

In a non-randomized feasibility study, Söderström et al. [57] examined whether a GFD had an influence on glycemic control in patients with T1DM. Children with recently diagnosed T1DM followed either a GFD or a normal diet for 12 months. The effect of GFD on glycemic control was analyzed by measuring c-peptide, HbA1c, and IDAA1c. Adherence to the GFD was also examined. At six months, a GFD resulted in a statistically significant lower HbA1c in comparison with a normal diet. At 6 and 12 months, there was better glycemic control in the GFD group. Adherence to a GFD varied but was deemed satisfactory by the majority of the children studied. Thus, a GFD can be adequately continued by children with new T1DM, and that may positively affect glycemic control.

In order to assess the effect of GFD on T1DM and subclinical CeD, Kaur et al. [58] conducted a randomized controlled trial. Patients with T1DM who had subclinical CeD were randomized to receive a normal diet or a GFD for 1 year. The study showed that there is a decrease in hypoglycemic episodes and better glycemic control in patients on a GFD. Further, the mean HbA1c decreased in the GFD group and increased in the group following a normal diet.

In another multicenter study [37], asymptomatic T1DM patients (8–45 years old) were screened for CeD. Biopsy-confirmed CeD participants were randomized to a GFD or a gluten-containing diet (GCD) to assess changes in HbA1c and continuous glucose monitoring over 12 months. Fifty-one participants were randomized to either a GFD (*n* = 27) or a GCD (*n* = 24). No HbA1c differences were seen between the groups (+0.14%, 1.5 mmol/mol; 95% CI: 0.79 to 1.08; *p* = 0.76), although greater postprandial glucose increases (4 h + 1.5 mmol/L; 95% CI: 0.4–2.7; *p* = 0.014) emerged with a GFD.

The high glycemic index of most GFD foods, complicates glycemic management in patients with T1DM and CeD. According to a comparison of different foods conducted by the American Society for Clinical Nutrition, the glycemic index of gluten-free foods is in most cases higher than that of gluten-containing equivalents. The glycemic index provides a good measure of carbohydrate absorption; therefore, ingestion of foods with a high glycemic index results in a more rapid rise in blood glucose values. The consequent hyperglycemia results in an increase in free fatty acids, which may induce oxidative stress and promote atherosclerosis [59,60].

In diabetic patients, a low glycemic index diet is often pursued to restrain glucose excursions that are associated with increased long-term risk of microvascular complications [61,62].

Because of the higher glycemic index of GFD, the blood glucose peaks in patients with T1DM and CeD appear earlier and are higher than in those without CeD [63]. Accordingly, the dose and timing of insulin administration need to be based on the nutrient content of the gluten-free products. Consumption of dietary items with a low glycemic index, such as, meat or vegetables, prior to the main source of carbohydrates may improve postprandial glycemic control and dampen potential fluctuations [64,65]. On the other hand, some gluten-free food items may be low in carbohydrates; hence, hypoglycemia may develop after the administration of standard (unadjusted) dosages of insulin. This emphasizes the necessity for carefully labeling food packages [64,65]. Table 1 presents an overview of literature on the effect of a GFD on glycemic control in patients with Celiac Disease and Type 1 Diabetes Mellitus.

### 3.3. Effect of Gluten Free Diet on Body Mass Index in Patients with T1DM and Celiac Disease

Data [52,66] on young patients following a GFD showed normal growth patterns in young patients with coexistent T1DM and CeD, with marginal but not significant deviations in BMI and height standard deviation scores in the non-celiacs. This adherence to GFD in young patients has no negative influence on HbA1c or insulin requirements. Factors that may predispose to an increased BMI are the ingestion of a diet containing carbohydrates with a high glycemic index, saturated fat, and a limited amount of proteins and fiber.

Intestinal absorption improves when mucosal healing occurs after eliminating gluten from the diet. This may result in significant weight gain, which may increase morbidity and the risk of cardiovascular disease [67], especially in T1DM patients. However, data on overweight in CeD patients are inconsistent. Dickey et al. [68] showed that, after 2 years on GFD, about 80% of CeD patients gained weight, and about 50% were overweight or obese. On the contrary, another study found that obese or overweight CeD patients lost weight while on GFD [69].

Decreased bone mineral density (BMD) is observed both in CeD [70,71] and T1DM patients [72].

### 3.4. Bone Mineral Density in Patients with T1DM and Celiac Disease

BMD in CeD + T1DM patients is generally decreased, and follow-up of BMD with eventual treatment is warranted. Besides following a strict GFD, supplementation of calcium and vitamin D is mandatory [73]. Lifestyle changes such as regular exercise, avoidance of excess alcohol, and smoking cessation should be advised, and in the case of documented osteoporosis, anti-osteoporosis medications should be considered [74].

## 4. Micro- or Macroangiopathic Complications in Coexistent T1DM and Celiac Disease

Few studies have been published dealing with the vascular complications of T1DM in the presence of CeD. Interestingly, Bakker et al. [75] showed that retinopathy is less prevalent in T1DM patients with CeD compared to controls (T1DM patients without CeD). This could imply that a GFD has a beneficial effect on vascular complications in T1DM patients.

Similar findings were observed by Picarelli et al. [76]. They studied whether CeD in patients with T1DM is associated with different expressions of hemostatic factors and if that is associated with an effect on the development of complications. They found that CeD may have a protective role in the prothrombotic state of T1DM. Patients with CeD had significantly lower cholesterol, triglycerides, HbA1c, factor VII antigen, coagulant activity, and prothrombin degradation fragments.

Contrary to these studies, Pitocco et al. [77] found that the carotid intima-media layer was thicker in T1DM patients with a long duration of CeD, compared to those diabetics without CeD. Data from the Diabetes Study Group of the Italian Society of Pediatric Endocrinology and Diabetology (ISPED) showed that the risk of cardiovascular disease in children with T1DM and untreated CeD may be increased by an unfavorable lipid profile (low HDL-C levels and high LDL-C values) [78]. These data underscore the fact that a strict GFD is mandatory for these young patients.

A multicenter longitudinal analysis from the German-Austrian DPV (Diabetes Patienten Verlaufsdokumentation) Database investigated whether CeD associated with T1DM increases the risk of microvascular complications [79]. Nephropathy and retinopathy occurred earlier in the presence of CeD. The incidence of retinopathy and nephropathy was higher in patients with T1DM and CeD than in those without CeD. This study did not investigate the possible protective effect of a GFD on the microvascular complications in T1DM and CeD.

In a case–control study [80] performed in Sheffield, U.K., T1DM patients aged > 16 years (*n* = 1000) were assessed for CeD. CeD was found in 3.3% of the study population. HbA1c, lipid profile, nephropathy stage, retinopathy stage, and degree of neuropathy before and after 1 year on a GFD were assessed. At the time of diagnosis of CeD, adult patients with T1DM had worse glycemic control, lower total cholesterol, lower HDL cholesterol, and a higher prevalence of retinopathy, nephropathy, and peripheral neuropathy. After following a GFD for one year, there was an improvement in the lipid profile, HbA1c, and markers for nephropathy. Of particular importance, if GFD has a protective role against the development of micro- and macroangiopathic complications, then a misdiagnosis of CeD in adult patients with T1DM would be associated with a higher prevalence of nephropathy, peripheral neuropathy, and retinopathy.

Creanzo et al. [56] found that the coexistence of T1DM and CeD is associated with lower eGFR values than those with T1DM alone.

These findings underscore the importance of regular screening for CeD in T1DM patients to timely detect those at risk of developing CeD.

On the other hand, GFD has been shown to have a protective rather than a detrimental effect on micro- and macrovascular complications [75,76], even in children [81,82].

To summarize, there seems to be greater agreement between studies on the benefits of GFD regarding long-term vascular complications. However, there are substantial differences between these studies, and consequently, the inconsistent and inconclusive results regarding the influence of a GFD on glycemic control, insulin dose, HbA1c, glucose excretion, and hypoglycemic episodes in patients with T1DM and CeD, they could be due to the type of diet that they actually follow. A GFD could mean the use of foods rich in fats with a high glycemic index and a more unfavorable impact, or it could mean a diet based mainly on vegetables with a more favorable impact on glycemic values, HbA1c, insulin requirement, lipid profiles, and even the incidence of long-term diabetic complications. Thus, investigating the ingredients of GFD would be more interesting.

Further investigations are needed to explore the mechanism by which therapy with a GFD might prevent micro- and macrovascular complications of T1DM. Theoretically, greater dietary adherence and regular assessment and coaching by an experienced dietitian might result in better and healthier eating habits. See Box 5.

Box 5 **Synopsis 5.**

1. CeD in T1DM patients is associated with increased risk of microvascular complications and worse macrovascular risk profile.

2. There is evidence suggesting GFD has a protective role in the development of micro-and macrovascular complications of diabetes mellitus rather than a deteriorating one.

3. Further investigations are needed to explore the mechanism by which therapy with a GFD might prevent micro- and macrovascular complications of T1DM.

Table 2 presents an overview of literature on micro- and macroangiopathic complications in coexistent diabetes mellitus and celiac disease.

## 5. Quality of Life

In general, coping with multiple chronic conditions may result in poorer health outcomes, an increased financial burden, and difficulties in social communication with others. Both CeD and T1DM are chronic illnesses that have an impact on quality of life (QoL) due to the cost of treatment and the complications associated with T1DM in particular. Families have more concerns about the complications of diabetes mellitus than do those with CeD. Families and young patients struggle with routinely measuring blood glucose levels and adhering to a strict GFD. Gluten-free products are usually expensive. Preparing foods and visits to clinics take significant amounts of time. Children may feel different from their peers and suffer from feelings of isolation and misunderstandings [83,84].

There are few reports assessing changes in health-related QoL after starting a GFD in patients with T1DM who are asymptomatic for CeD, as chronic conditions necessitate significant lifestyle changes. The study by Leeds et al. [80], mentioned earlier, found that treatment with a GFD for 1 year is safe in adults with T1DM and does not have a negative impact on QoL.

Sud et al. [85] performed a cross-sectional assessment study using a validated self-reported QoL measure: 28 children with biopsy-proven CeD and T1DM were compared with 40 subjects with T1DM aged 8–18 years. Parental and child reports were assessed regarding the quality of life as well as symptoms at two moments: the time of celiac disease diagnosis and at follow-up. No significant differences in quality of life were observed between subjects with established CeD and T1DM and subjects with T1DM alone. Parents of children with CeD and T1DM reported lower social functioning scores than parents of children with T1DM (*p* = 0.03). In the CeD and T1DM group, no differences in QoL were observed with regard to age at CeD diagnosis, CeD duration, or adherence to a GFD. Thus, it appears that having CeD in addition to T1DM has little effect on QoL; however, parents of CeD and T1DM children expressed greater concern about their child’s social functioning.

Weiman et al. [86] prospectively assessed QoL in patients with T1DM and asymptomatic CeD. They randomly assigned patients to GFD versus standard diet for 12 months. QoL was not significantly affected by following a GFD over 12 months; on the other hand, QoL worsened when HbA1c deteriorated and with the onset of symptoms. These findings suggest that adaptation to a GFD can be made successfully in this population without adversely affecting QoL Another, smaller study [57] found that following a GFD at inclusion negatively affected QoL, but the difference was not significant throughout the study.

A recent systematic review [66] concluded that adherence to GFD in young patients with T1DM and CeD results in improved QoL. See Box 6.

Box 6 **Synopsis 6.**

1. Coping with simultaneous multiple chronic conditions may result in impaired health outcomes, an increased financial burden, and difficulties in social communication with others.

However, it appears that the additional diagnosis of CeD has minimal impact on QoL in young patients with T1DM.

2. Parents of CeD + T1DM children expressed greater concern about their child’s social functioning.

## 6. Conclusions

Most patients with T1DM who develop CeD are either asymptomatic or only mildly symptomatic; less than 10% develop gastrointestinal symptoms. Patients affected by T1DM should undergo screening for asymptomatic CeD, particularly during the first 5 years after diagnosis, and potentially even thereafter. Both diseases are associated with an increased risk of developing other autoimmune disorders such as Hashimoto’s thyroiditis, Addison’s disease, and vitiligo.

The most challenging aspect for a child with T1DM and CeD is that most GFD foods have a high glycemic index. Interestingly, dietary therapy for CeD could improve the elevated HbA1c levels.

Avoiding gluten added to a diabetic dietary regimen in T1DM patients might impose practical limitations and lead to considerable restrictions in the lifestyle of a young patient. Consequently, non-adherence to GFD is common among patients with T1DM and CeD.

There is evidence suggesting GFD has a protective role in the development of micro- and macrovascular complications of diabetes mellitus. Theoretically, greater dietary awareness, increased attention to food intake, and regular assessment and coaching by a skilled dietitian should result in better control of carbohydrate intake and healthier eating habits.

It should be noted that there is a discrepancy between the data on overweight in CeD patients, and it is not yet known what the final effect of GFD on children with CeD in T1DM will be. Indeed, Dickey et al. [68] showed that after 2 years of GFD, about 80% of patients with CeD gained weight, and about 50% were overweight or obese. On the contrary, another study found that obese or overweight CeD patients lost weight while on GFD [69]. Thus, GFD certainly has positive effects, but it is necessary to understand how GFD helps improve the predictors of diabetes control.

It seems that adherence to GFD in young patients with T1DM and CeD leads to regular growth, without any negative effect on HbA1c or insulin requirements. Furthermore, although assessed in a few studies, the lipid profile may improve with the introduction of GFD without negatively impacting QoL.

## Figures and Tables

**Table 1:** Overview of literature on the effect of a gluten-free diet on glycemic control in patients with Celiac Disease and Type 1 Diabetes Mellitus.

References	Main Objective	Study Design	Study Population	Results	Comments and Study Limitations
Taler et al. 2012	Study the effect of CeD on glycemic control and growth in T1DM patients, and the influence of dietary adherence to GFD on these parameters.	Observational case–control study	68 T1DM patients and CeD	No significant differences in glycemic control or frequency of severe hypoglycemia or diabetic ketoacidosis between the study patients and controls. Standard deviations of Body mass index, height, and HbA1c values were not significantly higher in the control than the study group and similar in subjects with CeD regardless of degree of adherence to a GFD.	The numbers were small to make clear conclusions.
Acerini et al. 1998	Study the clinical characteristics and response to GFD in CeD in children and adolescents with T1DM	Screening for CeD	167 children and adolescents with T1DM (97 males; age 1.9–22 years) in a pediatric diabetic clinic	By 24 months, there was a trend towards increased BMI standard deviation score 1.31 (0.47 to 2.29), *p* = 0.248) and to reductions in HbA1c (8.1 (6.4–10.8), *p* = 0.697). The therapeutic benefits of dietary therapy in asymptomatic CeD remain uncertain.	No comment
Saadah et al. 2004	Study the effect of GFD on growth and diabetic control in children with T1DM and CeD.	Weight, height, Hb-A1c, and insulin requirements were measured before and for 12 months after the diagnosis and treatment of CeD. Dietary adherence and awareness were measured by structured questionnaire.	21 children with T1DM, and subsequent diagnosis of CeD.	BMI increased after GFD. Insulin dosage at diagnosis was less in CeD patients than in controls), but was similar to controls once a GFD had been established. It seemed that there was a relation between dietary adherence/awareness and growth parameters.	The small number of patients prevented relevant analysis.
Kaukinen et al. 1999	Study the effect of a GFD on metabolic control of diabetes in patients with T1DM and CeD. Additionally analyzed whether the diabetic control changes after reinforcement of GFD	Retrospective and controlled prospective survey. Published as a letter to the editor.	45 adults; 22 patients.	Retrospective analysis: there were no statistically significant differences in metabolic control, BMI, or hypoglycemic events before and after the introduction of the GFD. Prospective analysis: the mean HbA1c was 8.7% before and 8.8% after 1 year in patients with CeD. The mean daily insulin dosage was 0.6 U/kg in patients with CeD, and 0.7 U/kg in those with only T1DM. No differences were found in metabolic control, insulin dosage, BMI, and hypoglycemic events in CeD patients at the beginning and at the end of the study.	Very limited number of patients included.
Abid et al. 2011	Study the short-term effects of GFD in a T1DM and CeD.	Data were collected at baseline and 12 months after GFD.	468 children with T1DM; 23 patients were diagnosed with CeD	Almost 50% showed improvement in GI symptoms, while 75% of patients had no further severe hypoglycaemic events. There was no significant change in score for height, weight, and BMI or the mean HbA1c and before and after GFD. The mean insulin requirement increased.	The duration of study was short
Creanza et al. 2018	To study whether in T1DM patients the concomitant presence of CeD treated with a GFD influences glycemic control and the prevalence or severity of microvascular complications.	An observational case–control study	34 patients with T1DM and CeD and 66 patients with T1DM alone matched for age, gender, and T1DM duration	HbA1c level was similar in T1DM + CeD and T1DM alone; insulin requirement was significantly higher in T1DM + CeD compared with T1DM.	Observational and cross-sectional study design which prevented evaluation of the changes of renal function over time. Patients were followed up in a tertiary care center with periodic clinical assessment of both T1DM and CeD. Therefore, results may not be fitting to those who may not undergo a strict monitoring.
Söderström et al. 2022	Examing the effects of diet on glycemic control. Additionally examined adherence to GFD and effects on QoL	Non-randomized feasibility study	23 children with recent diagnosis of T1DM. A GFD (*n* = 14) or a normal diet (*n* = 9) for 12 months.	Children on a GFD had a statistically significant lower HbA1c at 6 months compared with those on a normal diet. Glycemic control was improved in the GFD group at 6 and 12 months. Adherence to a GFD was satisfactory. At inclusion, the GFD group had poorer QoL and there was no significant difference for QoL between groups throughout the study.	Small samples resulted in low statistical power. Participants were not randomized. The authors discussed these and other limitations extensively.
Mozzillo et al. 2022	Evaluate the influence of GFD on growth, metabolic control and QoL in young patients with T1DM and CeD.	Systematic review	Studies published in recent 15 years. Studies included were those having moderate-high quality of evidence and reporting objectively adherence to GFD	Studies showed no significant differences in growth parameters, HbA1c, number of hypoglycemic episodes, total daily insulin doses comparing youth with T1DM + CeD on GFD to those with T1DM only. Studies assessing the effect of GFD introduction showed stable BMI and HbA1c. Two studies assessed QoL, which was not different between T1DM + CeD versus T1DM only, as well as pre- and post-CeD diagnosis and introduction of GFD.	Heterogeneity of the analyzed studies regarding sample size, duration of follow-up, and methods.
Mahmud et al. 2020	Studied changes in HbA1c over 12 months after GFD	Randomized clinical trial	Fifty-one participants	51 patients were randomized to a GFD (*n* = 27) or Gluten containg diet (*n* = 24). No HbA1c differences were seen between the groups, although greater postprandial glucose increases emerged with a GFD.	Small sample size and heterogeneity of patients characteristics
Kaur et al. 2020	Assess the effect of GFD on T1DM and subclinical CeD	Prospective open label randomized controlled trial	320 T1DM patients. Thirty eligible patients were randomized to receive GFD (*n* = 15) or a normal diet (*n* = 15)	There is a decrease in hypoglycemic episodes and better glycemic control in patients on a GFD. Further, the mean HbA1c decreased in the GFD group and increased in the group following a normal diet.	-

**Table 2:** Overview of literature on micro- and macroangiopathic complications in coexistent diabetes mellitus and celiac disease.

References	Main Objective	Study Design	Study Population	Results	Study Limitations
Bakker 2013	To study 1. the glycemic control at CeD diagnosis and after initiating a GFD in T1DM patients. 2. the prevalence of complications in T1DM patients with CeD.	Retrospectively collected HbA1c levels before CeD diagnosis, at CeD diagnosis, and the latest HbA1c levels as well as the presence of nephropathy and retinopathy.	31 patients; median duration of T1DM and CeD of 27 years (IQR 14–37) and 3 years (IQR 1–8), respectively.	Prevalence of retinopathy was lower in T1DM + CeD group compared with controls, (38.7 vs. 67.4%, *p* < 0.05), no difference in the prevalence of nephropathy was found between the groups (*p* = 0.09).	A retrospective, observational study, and therefore associations may not reflect causality.
Picarelli 2013	Evaluating whether the presence of CeD in a group of T1DM patients is associated with a different expression of hemostatic factors and with a different manifestation and/or progression of microvascular complications of T1DM in comparison with patients with only diabetes	Laboratory assessment of blood and urine samples	94 adult T1DM patients were included and subsequently screened for CeD.	The metabolic control and the hemocoagulative parameters were significantly different between the two groups: T1DM + CeD patients had significantly lower concentrations of HbA1c, cholesterol, triglycerides, factor VII antigen, factor VII coagulant activity (FVII:c), and prothrombin degradation fragments, as well as higher values of activated C protein. Neither retinal abnormalities nor signs of renal damage were observed in T1DM + CeD patients.	-
Pitocco 2011	To assess carotid intima-media thickness (c-IMT), in patients with T1DM, CeD or both (T1DM + CeD) compared with age- and sex-matched healthy individuals.	Observational single centre study. Clinical, metabolic and anthropometric data were collected.	120 patients, 30 with T1DM, 30 with CeD, 30 with T1DM + CeD and 30 Healthy controls.	c-IMT was significantly greater in patients with T1DM + CeD than in those with T1DM or CeD, while no difference was found between T1DM and CeD. c-IMT was greater in CeD than in healthy controls. Glycemic control was similar between T1DM + CeD and T1DM alone. Lipid and anthropometric parameters were similar among groups. In a multivariate analysis, only age and disease type were significantly correlated with c-IMT.	Observational study
Salardi 2017	To evaluate lipid profiles, besides HDL-C, in children with T1DM associated with biopsy-proven CeD, and to investigate the influence of age and degree of adherence to GFD on lipid changes.	Retrospective multicenter study	261 children with both T1DM and CeD were enrolled	At CeD diagnosis, children with T1DM + CeD showed higher LDL cholesterol (LDL-C) compared to children with T1DM alone. GFD failed to normalize LDL-C levels. HbA1c values were not affected by GFD. At diagnosis, the youngest children had lower levels of total cholesterol. These patients had a greater decrease in triglycerides levels after treatment.	No reliable data on complications, to relate them with lipid panel. Furthermore, The assessment of complications was not centralized.
Rohrer 2015	To investigate whether CeD associated with T1DM increases the risk of microvascular complications.	A Multicenter Longitudinal Analysis	Patients (*n* = 56,514) aged > 10 years with diabetes duration < 20 years from 392 centers in Germany and Austria,	Retinopathy and nephropathy occurred earlier in the presence versus absence of CeD: retinopathy was seen at mean age 26.7 years in 25% of patients with CeD vs. mean age 33.7 years in 25% without CeD. Microalbuminuria was documented at mean age 32.8 years vs. 42.4 years. The hazard ratio both retinopathy (1.263) and nephropathy (1.359) was higher in patients with T1DM and CeD versus those without CeD. After adjustment for confounders, CeD was found as an independent risk factor for microvascular complications.	-
Leeds 2011	Investigated the effect of a GFD on diabetes-related complications in CeD in adults with T1DM.	A case-control study conducted at a U.K. teaching hospital.	Patients with T1DM aged > 16 years (*n* = 1000) were assessed for CeD. HbA(1c), lipid profile, quality of life, retinopathy stage, nephropathy stage, and degree of neuropathy before and after 1 year on a GFD were assessed.	The prevalence of CeD was 3.3%. At diagnosis of CeD, adult T1DM patients had worse glycemic control, lower total cholesterol, lower HDL cholesterol, and a higher prevalence of retinopathy, nephropathy, and peripheral neuropathy. There was no difference in QoL (*p* > 0.1). After 1 year on a GFD, only the lipid profile improved, but in those adherent to GFD HbA1c and markers for nephropathy improved.	-
Creanza 2018	To study whether in T1DM patients the concomitant presence of CeD treated with a GFD influences glycemic control and the prevalence or severity of microvascular complications.	An observational case-control study	34 patients with T1DM and CeD and 66 patients with T1DM alone matched for age, gender, and T1DM duration.	HbA1c level was similar in T1DM + CeD and T1DM alone; insulin requirement was significantly higher in T1DM + CeD compared with T1DM.	Observational and cross-sectional study design which prevented evaluation of the changes of renal function over time. Patients were followed up in a tertiary care center with periodic clinical assessment of both T1DM and CeD. Therefore, results may not be fitting to those who may not undergo a strict monitoring.

## Data Availability

Not applicable.
